# Immune Cell Dynamics in EGFR-Mutated NSCLC Treated With Afatinib and Pembrolizumab: Results From a Phase IB Study

**DOI:** 10.1016/j.jtocrr.2024.100706

**Published:** 2024-07-14

**Authors:** Jonathan W. Riess, Matthew S. Lara, Miguel Lopez de Rodas, Guillaume Luxardi, Samantha Herbert, Michiko Shimoda, Karen Kelly, Alexander Meerlev, Elizabeth Moore, Laurel Beckett, Arta Monjazeb, Kurt Schalper, Emanual Maverakis, David R. Gandara

**Affiliations:** a University of California Davis Comprehensive Cancer Center, Sacramento, California; b Yale School of Medicine and Yale Cancer Center, New Haven, Connecticut

**Keywords:** Lung cancer, Epidermal growth factor, Immunotherapy, Targeted therapy

## Abstract

**Introduction:**

EGFR-mutated NSCLC is minimally responsive to programmed cell death protein 1 or programmed death-ligand 1 blockade. We evaluated the safety, tolerability, and immunomodulatory effects of the EGFR tyrosine kinase inhibitor (TKI) afatinib in combination with the programmed cell death protein 1 antibody pembrolizumab in patients with EGFR-mutant NSCLC.

**Methods:**

Patients with advanced EGFR-mutant NSCLC with progression (PD) on previous EGFR TKI(s), aged above or equal to 18 years, Eastern Cooperative Oncology Group performance status less than or equal to 1, acceptable organ function, no significant autoimmune disease, measurable disease, and controlled brain metastases were eligible. Primary end point was determination of the maximum tolerated dose and recommended phase 2 dose. Serial specimens were collected to assess for alterations in cytokines and immune cell subsets by quantitative immunofluorescence in tissue and Luminex and flow cytometry in the blood.

**Results:**

A total of 11 patients were enrolled, six in dose finding and five in dose expansion. No dose-limiting toxicities were observed. The maximum tolerated dose was determined to be afatinib 40 mg orally daily and pembrolizumab 200 mg intravenously every 21 days. Four (36%) patients had immune-related adverse events (irAEs). Ten patients were assessable for response: two partial response, seven stable disease, and one PD. Peripheral natural killer and natural killer T-cells (*p* = 0.027, *p* = 0.01) increased and exhausted CD8+ T-cells decreased on treatment (*p* = 0.0035). Peripheral CD4/CD8 T-cells (area under the curve = 0.96, *p* = 0.042) and central memory T-cells (CD4/CD8) (area under the curve = 1.0, *p* = 0.0006) increased in patients who had disease control more than 6 months or partial response to afatinib/pembrolizumab as did CD3+ T-cells in a patient with progression-free survival more than 6 months after afatinib/pembrolizumab treatment.

**Conclusions:**

Afatinib and pembrolizumab were found to have modest activity associated with irAEs after PD on previous EGFR TKI setting. Proinflammatory changes in immune cell subsets in tissue and blood were detected and associated with antitumor activity and irAEs.

## Introduction

Lung cancer is the leading cause of cancer deaths worldwide and often presents as incurable metastatic disease. The development of targeted therapies, such as EGFR tyrosine kinase inhibitors (EGFR TKIs), has greatly advanced lung cancer treatment.^1^ Similarly, programmed cell death protein 1 (PD-1) or programmed death-ligand 1 (PD-L1) immune checkpoint inhibitors (ICIs) have revolutionized the treatment of NSCLC and are approved as both single agents and in combination with chemotherapy plus or minus CTLA-4 inhibitors in NSCLC.^2^, ^3^, ^4^, ^5^ Nevertheless, lung cancers harboring EGFR-activating mutations are generally insensitive to immune checkpoint blockade, though anti-angiogenesis agents may potentiate ICI activity in EGFR-mutated NSCLC.^6^^,^^7^

Preclinical studies suggest that mutant EGFR may play some role in altered regulation of PD-L1 expression and the immune microenvironment and that EGFR TKIs may modulate the immune microenvironment, spurring multiple clinical trials combining EGFR TKI and PD(L)1 antibodies.^8^ For example, a phase 1 clinical trial of nivolumab combined with erlotinib reported moderate antitumor activity.^9^

Nevertheless, there have been concerning observations regarding potentiation of adverse events (AEs) when first- or third-generation EGFR TKIs are administered with immunotherapy, including pneumonitis in patients treated with PD-(L)1 antibodies and osimertinib.^10^^,^^11^ Increased rates of hepatitis were observed with the gefitinib/durvalumab and erlotinib/atezolizumab combinations.^12^^,^^13^ Overall, AEs seem to be magnified when EGFR TKIs are administered with PD-(L)1 antibodies.^14^

Afatinib is a small-molecule, selective, and irreversible erbB family blocker. It is a second-generation EGFR TKI approved for the treatment of NSCLC for common (E19del and L858R) and uncommon (G719X, L861Q, and S768I) EGFR mutations and for patients with advanced squamous NSCLC after progression on previous therapy.^15^^,^^16^ It is tolerable as a single agent, with the main adverse effects being diarrhea, rash or acne, and stomatitis. Afatinib improves progression-free survival (PFS) in patients with advanced EGFR-mutant NSCLC compared with chemotherapy as first-line treatment and as second-line treatment after EGFR TKI has an overall response rate (ORR) of approximately 5% to 8% and median PFS (mPFS) of 3.3 to 4.4 months after other EGFR TKI treatment.^17^, ^18^, ^19^

Enhancing immunotherapy efficacy in EGFR-mutant NSCLC, where single-agent ICI activity is limited, represents a major unmet need. Afatinib has been found to enhance CD8+ T-cell effectiveness. A study using MC38 colon cancer xenograft models revealed that combination of afatinib and PD-1 blockade was more effective in inhibiting tumor growth than either of the single agent alone.^20^ Moreover, independent of its activity against mutant EGFR, afatinib has been found to potently enhance antigen-specific cytotoxic T-lymphocyte killing.^20^

In this prospective, nonrandomized, open-label, phase I clinical trial, we sought to assess the safety and preliminary efficacy of combination afatinib and pembrolizumab in patients with advanced NSCLC with EGFR-activating mutations who have experienced disease progression on previous EGFR TKI. We also sought to evaluate alterations in circulating immune cells and the tumor immune microenvironment in patients who received afatinib and pembrolizumab on this clinical trial.

## Methods

### Eligibility

This study was a prospective, nonrandomized, open-label, phase I clinical trial conducted at the UC Davis Comprehensive Cancer Center that enrolled patients from 2015 to 2018. To be eligible, patients aged 18 or older had to have metastatic or recurrent NSCLC harboring an EGFR-activating mutation (exon 19 del, exon 21 L858R, L861Q, G719X, S768I) and have progressive disease on erlotinib or gefitinib or osimertinib. There was no limitation to previous lines of treatment. Eligible patients must have had a life expectancy of more than or equal to 3 months and have adequate archival tissue or consent to a fresh tissue biopsy at baseline and fresh biopsy within 3 days of cycle 3, day 1 treatment. Patients were required to have adequate end-organ function and a performance status of 0 or 1 on the Eastern Cooperative Oncology Group (ECOG) Performance Scale.

The study was approved by the UC Davis Institutional Review Board. All patients were required to provide written informed consent before participating, and all procedures were undertaken in accordance with the Declaration of Helsinki.

### Study Design and Treatment

This is a phase I/Ib clinical trial that tested the combination of afatinib and pembrolizumab in patients with advanced EGFR-mutant NSCLC with progression on previous EGFR TKI therapy (trial schema found in Supplementary Fig. 1). Cycle length was 3 weeks. Pembrolizumab dose was fixed at 200 mg intravenously (IV) every 3 weeks. For the dose de-escalation phase, afatinib was started at dose level 0 (40 mg daily). De-escalation followed a standard 3 + 3 design. This phase was to establish a maximum tolerated dose (MTD) of the combination for subsequent dose expansion.

There were two planned dose expansion cohorts. Patients on arm A were to receive concurrent afatinib and pembrolizumab with no lead-in treatment, whereas patients on arm B were to receive lead-in pembrolizumab followed by adding afatinib to pembrolizumab at the start of cycle 2. The MTD was to be further explored in these cohorts to determine the recommended phase II dose (RP2D), assess preliminary efficacy, and evaluate immune correlative studies. By protocol amendment, owing to the changing landscape of treatment in EGFR-mutant NSCLC, the study was closed early and accrued patients only on dose escalation with concurrent afatinib and pembrolizumab and arm A dose expansion (also concurrent afatinib and pembrolizumab) (Supplementary Fig. 1).

### End Points and Statistical Design

Analyses were limited to patients who received treatment. Three patients were initially entered at dose level 0. If zero of three or one of three patients experienced dose-limiting toxicity (DLT) at this (or any) dose level, an additional three patients were accrued. If two or more DLTs occurred at any dose level, three patients were then entered at the next lower dose level, until the MTD was determined. Two or more DLTs at a dose level would require termination of the study, whereas one DLT of six at dose level -2 would establish the MTD for dose expansion. All patients who did not experience a DLT were observed for a minimum of 21 days or until completion of their first cycle of therapy.

All toxicities were graded using National Cancer Institute (NCI) CTCAE version 4.0. The occurrence of any of the following toxicities during cycle 1 was considered a DLT, if judged by the investigator to be possibly, probably, or definitely related to study drug (afatinib plus or minus pembrolizumab) and occurring within 21 days or until their completion of first cycle of treatment: Grade 4 nonhematologic toxicity, grade 4 hematologic toxicity lasting more than or equal to 14 days. Grade 3 nonhematologic toxicity (not laboratory) lasting more than 3 days despite optimal supportive care. Any grade 3 or grade 4 nonhematologic value if: medical intervention is required, the abnormality leads to hospitalization, or the abnormality persists as more than or equal to grade 3 for more than 1 week. Febrile neutropenia grade 3 or greater. Thrombocytopenia less than 25,000/mm^3^ if life threatening or requiring a transfusion or a grade 5 toxicity (i.e., death).

Diarrhea and rash are expected AEs from afatinib. Diarrhea attributed to afatinib was considered a DLT if grade 3 despite maximal medical management for more than 72 hours. Grade 4 diarrhea or rash was considered a DLT. To be assessable for a DLT, 80% of the dose must have been administered in cycle 1 unless a DLT occurred. Delay in starting cycle 2 of more than or equal to 14 days due to toxicity related to afatinib plus or minus pembrolizumab was also considered a DLT.

The baseline assumption was that single-agent afatinib yields a response rate of 10%, and a response rate of 10% or less would not warrant further study. A response rate of more than or equal to 40% would justify further study. With 10 patients enrolled in cohort A expansion arm, we declared this combination lacking promise if fewer than three patients respond, and potentially worthy of further study if three or more patients respond. If the true response rate was 10%, the chance of having more than or equal to three patients respond is 7%. If the true response rate is more than or equal to 40%, the chance of having more than or equal to three patients respond was 83%.

### Peripheral Blood Collection and Analyses

Blood samples for correlative analysis were drawn at baseline, at cycle 3 day 1, and at progression. Two analyses on peripheral blood were undertaken: (1) multicolor flow cytometry of peripheral blood mononuclear cells (PBMC) to identify changes in immune subsets and (2) plasma protein levels at baseline were analyzed using the Multiplex Bead-based Luminex platform for multianalyte detection of plasma cytokines as described in previous publications.^21^^,^^22^ Post hoc comparisons stratified by patients who had a partial response to treatment or more than 6-month PFS were conducted.^23^ Receiver operator characteristic curve was constructed and the area under the curves (AUCs) were calculated for immune cell populations of interest and outcomes of clinical benefit.

### Tissue PD-L1 Immunohistochemistry and Multiplexed Immunofluorescence Staining

Tissue biopsies were performed at baseline and within 7 days of cycle 3 day 1. Using multiplex quantitative immunofluorescence, we measured the levels of tumor-infiltrating lymphocytes (DAPI/Cytokeratin/CD8/CD4/CD20), immune inhibitory receptors (DAPI/CD3/LAG-3/PD-1/TIM-3), and activation markers (DAPI/Cytokeratin/CD3/Ki-67/GrzB). Validation and in-depth details about the staining protocols have been published elsewhere.^24^^,^^25^ The levels of immune markers stained with multiplexed immunofluorescence were scored using the AQUA method of automated immunofluorescence using spatial molecular compartments and co-localization strategies. PD-L1 protein expression was performed by conventional chromogenic immunohistochemistry using a clinical-grade assay (22C3) and scored in a semiquantitative fashion using tumor proportion score (TPS) by a pathologist using light microscopy.

## Results

### Patients

A total of 11 patients were enrolled on this trial. Median age was 69 years. There were four male (36%) and seven female (64%) participants. All patients had NSCLC harboring EGFR-activating mutations. Six patients (54%) had Ex19del mutations, four patients (36%) had L858R, and one patient (9%) had a L861Q mutation. Nine patients (82%) had adenocarcinoma, one patient (9%) had squamous cell carcinoma, and one patient (9%) had a non–small cell neuroendocrine transformation. Six patients (54%) had previous erlotinib monotherapy, whereas five patients (46%) had previous erlotinib and osimertinib therapy. Furthermore, eight patients (73%) had tumors that were PD-L1 positive, two patients (18%) had insufficient tumor to determine baseline PD-L1 expression, and one patient (9%) had no PD-L1 expression (Table 1).

**Table 1:** Summary of Key Clinical, Pathologic, and Molecular Characteristics and Outcomes of Study Patients

Pt ID	Age	Gender	Histology	EGFR Mutation	Resistance Mechanism	Prior EGFR TKI	Baseline PD-L1 Expression (22C3)	Best Response	PFS (d)	irAE
UCD-001	82	M	Squamous	E19del	Unknown	Erlotinib	40%	PR	344	Yes (G2 nephritis)
UCD-002	65	M	Adenocarcinoma	E19del	MET amp	Erlotinib	20%	SD	81	No
UCD-003	39	F	Adenocarcinoma	E19del	Unknown	Erlotinib	QNS	SD	27	No
UCD-004	83	M	Adenocarcinoma	L858R	T790M	Erlotinib	1%	SD	510	Yes (G2 adrenal insufficiency)
UCD-005	47	M	Neuroendocrine Carcinoma	L858R	HER2 amp (neuroendocrine)	Erlotinib	0%	PD	35	No
UCD-006	75	F	Adenocarcinoma	E19del	T790M	Erlotinib, osimertinib	10%	SD	77	No
UCD-007	69	F	Adenocarcinoma	L861Q	Unknown	Erlotinib	25%	SD	186	Yes (G3 colitis)
UCD-008	53	F	Adenocarcinoma	E19del	T790M	Erlotinib, osimertinib	75%	PD	7	No
UCD-009	70	F	Adenocarcinoma	E19del	T790M/C797S	Erlotinib, osimertinib	30%	PD	8	No
UCD-010	76	F	Adenocarcinoma	L858R	Unknown	Erlotinib, osimertinib	QNS	SD	84	No
UCD-011	62	F	Adenocarcinoma	L858R	MET amp	Erlotinib	90%	PR	> 42	Yes (G3 colitis)

G, grade; PD, progressive disease; PD-L1, programmed death-ligand 1; PFS, progression-free survival; PR, partial response; SD, stable disease; TKI, tyrosine kinase inhibitor.

### Safety

Six patients were enrolled in the dose-finding portion of the study at dose level 0 (concurrent afatinib 40 mg orally daily and pembrolizumab 200 mg IV every 3 wk). No DLTs were observed in this group. Five patients were subsequently enrolled in the expansion cohort A with concurrent afatinib and pembrolizumab (Table 1). Dose levels were the same in dose escalation and dose expansion. The study was subsequently closed due to the changing landscape of treatment for advanced EGFR-mutated lung cancer. One patient was hospitalized within 7 days of study dosing due to progressive disease and came off study and was unassessable. Diarrhea (90%) and acneiform rash (60%) were the most common treatment-related AEs consistent with the known side effect profile of afatinib. Four patients (36%) had immune-related adverse events (irAEs): grade 2 adrenal insufficiency, grade 2 nephritis, and two grade 3 colitis events. All these patients either had a partial response or were progression free for more than or equal to 6 months but eventually discontinued study drugs because of toxicity. The median number of treatment cycles was 2 (range: 1–4). The toxicity data are summarized in Table 2.

**Table 2:** Adverse Events Occurring More Than 10% and All-Grade 3 to 4 Adverse Events Possibly, Probably, or Definitely Related to Study Drugs Graded by CTCAE Version 4 (N = 10)

Treatment-Related Adverse Event	All (%)	Grades 3–4
Diarrhea	9 (90)	2 (20%)
Rash acneiform	6 (60)	0
Lymphocyte count decreased	5 (50)	1 (10%)
Anorexia	5 (50)	1 (10%)
Fatigue	4 (40)	0
Creatinine increased	4 (40)	0
Anemia	4 (40)	0
Nausea	4 (40)	0
Hypoalbuminemia	3 (30)	0
Weight loss	3 (30)	0
Dry skin	3 (30)	0
Hypokalemia	3 (30	1 (10%)
Colitis	2 (20)	2 (20%)
Paronychia	2 (20)	0
Headache	2 (20)	0
Mucositis	2 (20)	0
Pruritus	2 (20)	0
Elevated liver function tests	2 (20)	0
Vomiting	2 (20)	0

### Efficacy

Ten patients were assessable for response; of these, two had a partial response, six had stable disease, and two had progressive disease (Table 1). Of those patients with stable disease, six had some degree of tumor size reduction not meeting Response Evaluation Criteria in Solid Tumors (RECIST) 1.1 criteria for response (Supplementary Fig. 2). Overall, four patients experienced either a partial response or PFS more than or equal to 6 months. All four of these patients experienced irAEs. One responding patient had squamous histology, previous progression on erlotinib, PD-L1 TPS 40%, PD-L1 amplification on next-generation sequencing (NGS), and a PFS of 11 months (Supplementary Fig. 2). The other patient with partial response (PR) had MET amplification on progression on 1L erlotinib and PD-L1 expression of 90% TPS. Both patients who responded to treatment came off study for irAEs (G2 nephritis and G3 colitis, respectively). One patient developed symptomatic rapidly progressive disease and was taken off study after 7 days.

### Immune and Molecular Correlative Studies

A key aim of this trial was to explore the immunomodulatory effects of combination EGFR TKI and PD-1 therapy on both circulating immune cells and the tumor immune microenvironment. Both blood and tissue samples were collected for this purpose.

**Figure 1:**
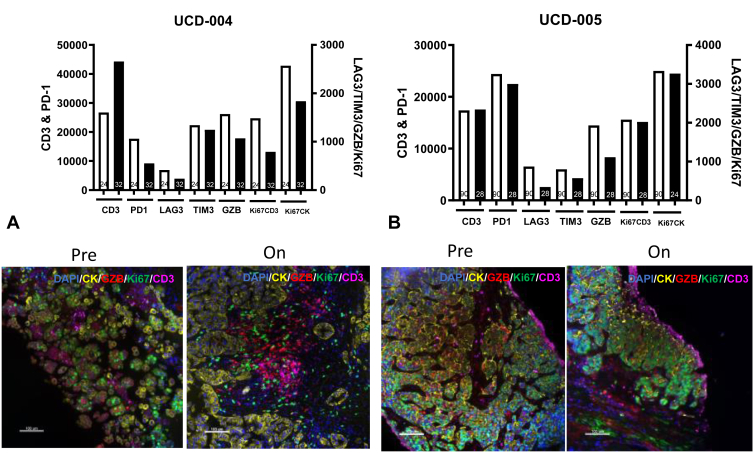
Quantitative immunofluorescence detects changes in the immune microenvironment in patients with advanced EGFR-mutant NSCLC treated with afatinib and pembrolizumab. (*A*) Patient with 20% tumor shrinkage and PFS more than 6 months. Increase in CD3+ T-cells and decrease in tumor proliferation (Ki67) noted. (*B*) Patient with progression at first interval imaging. No change in CD3+ T-cells or tumor proliferation (Ki67). PFS, progression-free survival.

Tissue samples were analyzed using quantitative immunofluorescence (QIF) of immune proteins. Seven patients at baseline had on-treatment biopsies or adequate archival tissue for QIF. Four of 10 assessable patients had repeat on-treatment biopsies (40%) with two patients having adequate tissue for QIF. Representative QIF analysis from tumors obtained as part of serial biopsies in two patients who also had on-treatment biopsies with adequate tissue—one who had clinical benefit and another who had progression—is found in Figure 1. In the patient who derived clinical benefit from afatinib/pembrolizumab, proinflammatory changes in the tumor immune microenvironment were observed. In addition, this patient who experienced a 20% tumor reduction and PFS more than 1 year had an approximately 40% increase in CD3+ T-cells and a marked decrease in cancer-cell Ki-67, indicating a reduction in tumor proliferation. Slightly lower levels of T-cell immune inhibitory receptors PD-1, LAG-3, and TIM-3, proliferation, and GZMB were also observed in this patient’s tumor supporting reduced T-cell exhaustion/dysfunction during treatment. A higher CD4/CD8 ratio in the tumor microenvironment was also noted in the patient who experienced clinical benefit to the combination with a numerically higher CD4/CD8 ratio in patients with PFS more than 6 months or PR to afatinib/pembrolizumab but was not statistically significant (*p* = 0.4) with a small sample size (N = 7) (Supplementary Fig. 3). Most of these changes were not found in the tumor microenvironment of a patient who had adequate tissue in an on-treatment biopsy and experienced rapid disease progression (Fig. 1).

To monitor the immunologic effects of afatinib and pembrolizumab on the peripheral immune system, multianalyte detection of plasma cytokines using Luminex (N = 8) and high-parameter flow cytometry was performed at baseline (N = 11), after cycle 3 of therapy (N = 4) and at progression (N = 5). Plasma was available for eight patients for detection of plasma cytokines using Luminex with no meaningful differences detected (data not revealed). Nevertheless, we detected noteworthy findings in peripheral blood immune cell populations.

### Treatment With Afatinib and Pembrolizumab Is Associated With Increases in NK and NKT Cells and a Decrease in Exhausted Cd8+ T-Cells

**Figure 2:**
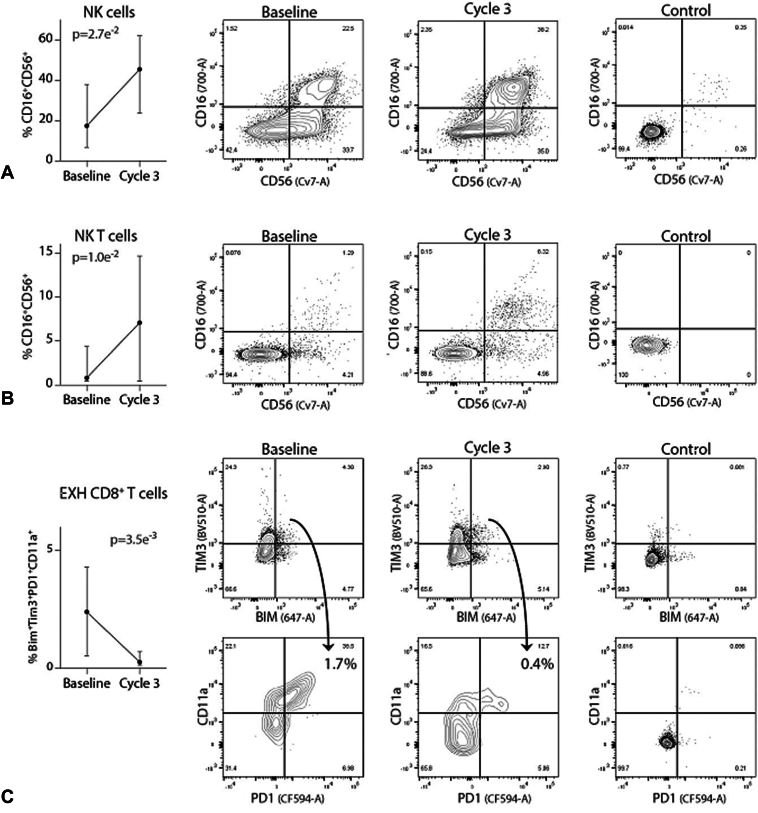
Flow cytometric analysis of immune cell frequencies. Treatment-induced trends in NK cell subpopulations over time. (*A*) NK cell frequency (CD3^−^/CD16^+^/CD56^+^) increases between baseline and cycle 3. Representative flow cytometry contour plots representing data used to construct line graph are revealed. (*B*) NKT cell frequency (CD3^+^/CD16^+^/CD56^+^) between baseline and cycle 3. (*C*) Exhausted T-cell frequency (Bim+Tim3+PD1+CD11a+CD8+) between baseline and cycle 3. Median values with 75th and 25th percentile whiskers. EXH, exhausted; NK, natural killer; paired Student’s *t* test.

Analysis of lymphocyte populations revealed that afatinib and pembrolizumab therapy was associated with significant increases in both NK and NKT cell populations (Fig. 2*A* and *B*, *p* = 0.0027 and *p* = 0.01, respectively). In contrast, treatment was associated with a decrease in circulating exhausted CD8+ T-cells at cycle 3 (Fig. 2*C*, *p* = 0.0035).

### Baseline Percentage Cytotoxic Effector Memory T-Cells Can Predict Response to Therapy

**Figure 3:**
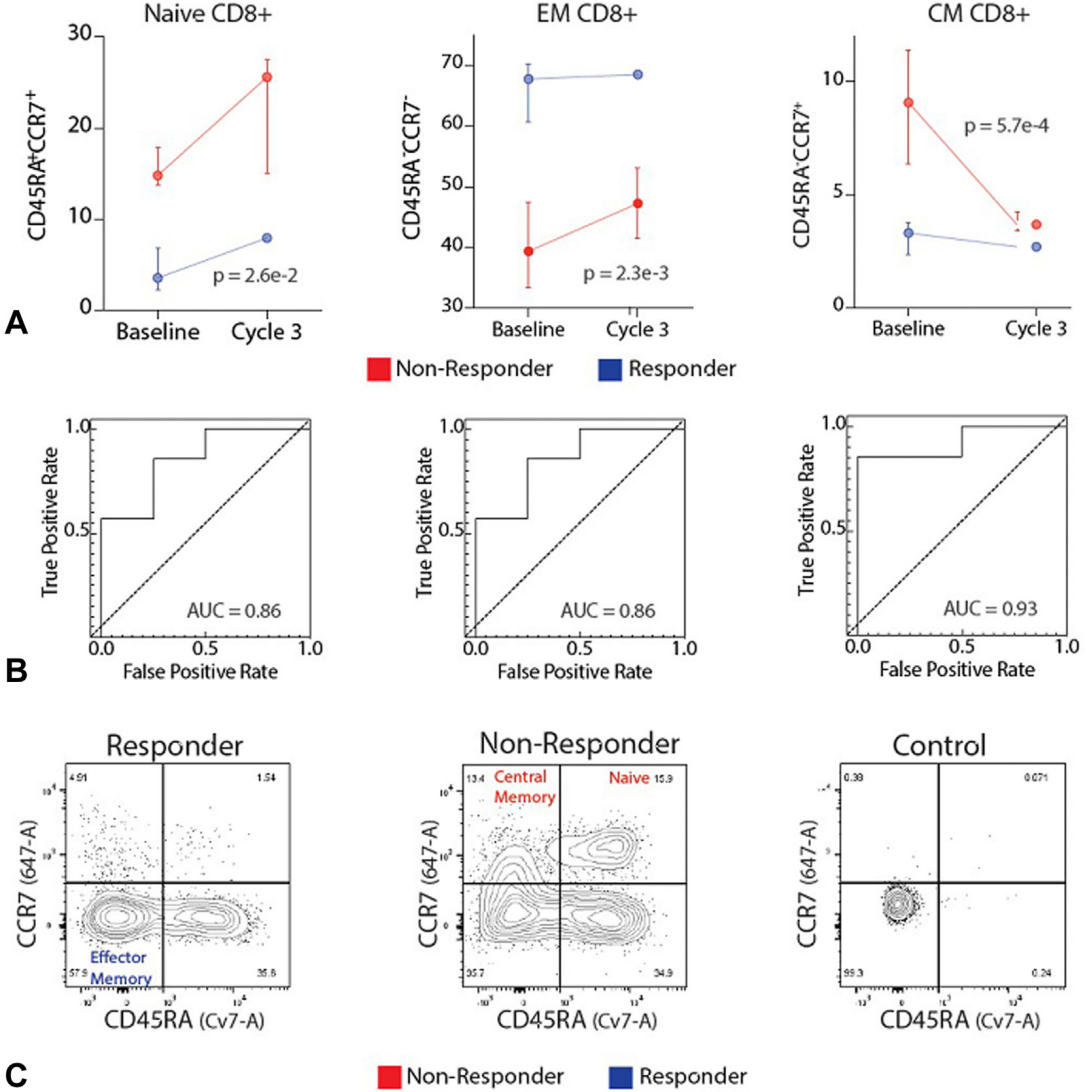
Baseline immune cell frequencies predict antitumor activity and irAEs on afatinib and pembrolizumab. Flow cytometric analysis from patients with disease control more than 6 months or PR to treatment (R) (blue) versus patients with PD or PFS less than 6 months (NR) (red). (*A*) Line graphs revealing frequencies of CD8^+^ effector memory T-cells, naive CD8^+^ T-cells, and CD8^+^ central memory T-cells at baseline and day 1 of cycle 3. Median values with 75th and 25th percentile whiskers. A mixed effects model was used to calculate the repeated measures *p* value. (*B*) Receiver operator characteristic curve for baseline CD8^+^ effector memory T-cells, naive CD8^+^ T-cells, and CD8^+^ central memory T-cells, R versus NR. Corresponding AUC are revealed. (*C*) Representative flow cytometry contour plots representing data used to construct line graph and receiver operator characteristic curves are revealed. Note: All patients with disease control more than 6 months or PR to treatment also experienced an irAE. Nonresponders did not experience irAEs. AUC, area under the curve; CM, central memory; EM, effector memory; irAE, immune-related adverse event; PFS, progression-free survival.

Cytotoxic (CD8+) T-cells can recognize and kill malignant cells. Previous studies across multiple tumor types have revealed that high baseline tumor-infiltrating T-cells are associated with improved clinical outcomes.^26^ We thus sought to assess the utility of the different CD8+ T-cell subpopulations to classify response to therapy using peripheral blood cell analysis. The results reveal that effector memory CD8+ T-cells were significantly higher at baseline and before initiation of cycle 3 in patients who ultimately would have a response or lengthy PFS to therapy (*p* = 0.0023, mixed effects model, Fig. 3*A*). Effector memory CD8+ T-cells (the percentage among all CD8+ T-cells) also predicted response to therapy—separating responders from nonresponders (AUC = 0.86 at baseline, Fig. 3*B*).

In contrast to cytotoxic effector memory CD8+ T-cells, naive CD8+ T-cells and central memory CD8+ T-cells (percentage of all CD8+ T-cells) were significantly decreased in patients who had a response or PFS more than 6 months versus nonresponders (*p* = 0.026 and *p* = 0.00057, mixed effects model, Fig. 3*B* and *C*). To evaluate the ability of these T-cell subpopulations to classify patients with antitumor benefit to afatinib/pembrolizumab from those with early progression or lack of response to treatment, receiver operator characteristic curves were constructed and AUCs were calculated (AUC = 0.86 and 0.93, respectively, at baseline; Fig. 3*B* and *C*).

### Cell Ratios That Predict Response to Therapy

**Figure 4:**
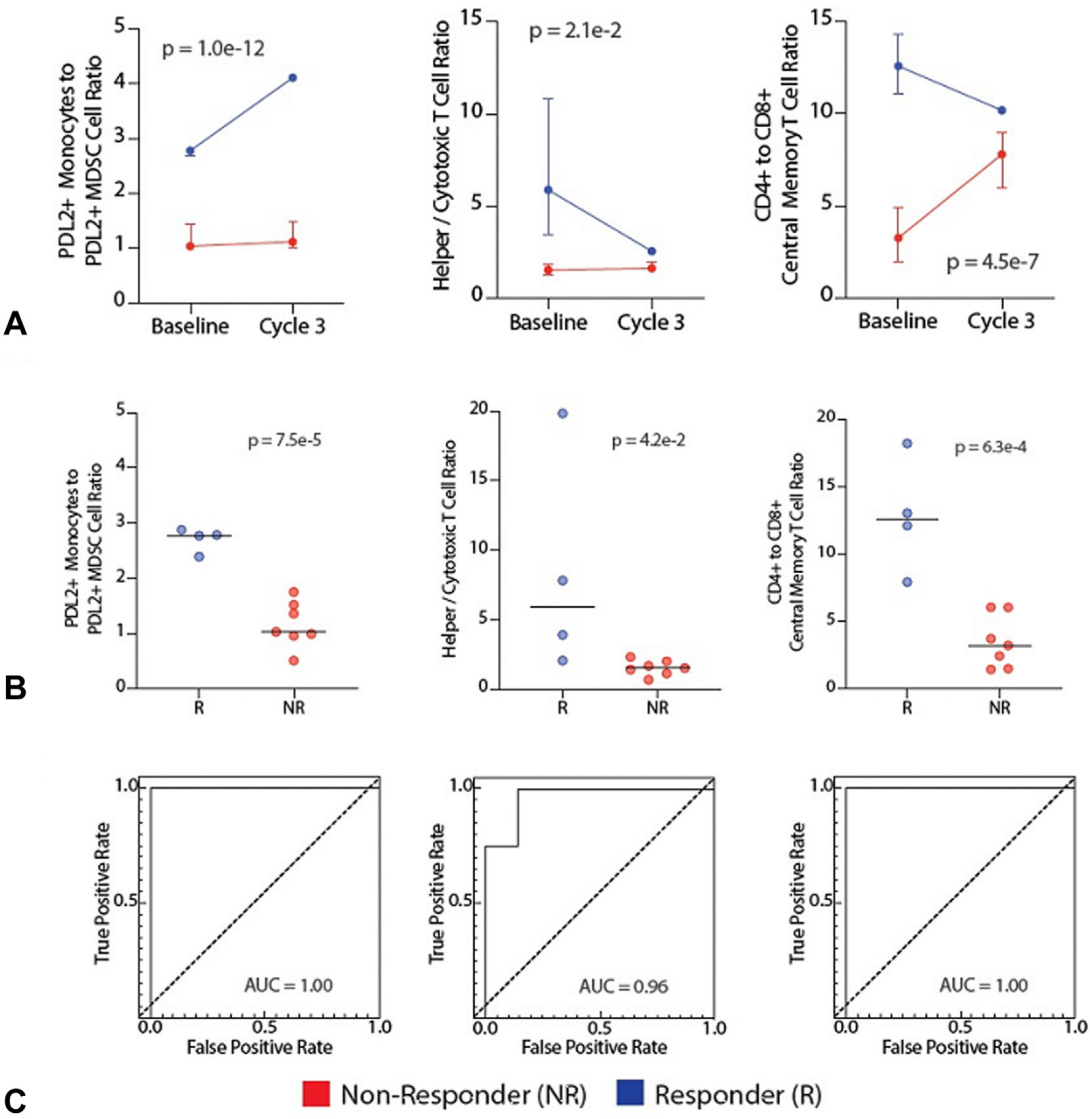
Flow cytometric analysis of cell frequency ratios. Flow cytometric analysis in patients with disease control more than 6 months or PR to treatment (R) (blue) versus patients with PD or PFS less than 6 months (NR) (red). (*A*) Line graphs indicate PDL2^+^ monocyte to PDL2^+^ MDSC, CD4 to CD8 T-cell and CD4^+^ to CD8^+^ central memory T-cell ratios are significantly different in R and NR. Line graph represents values at baseline and after initiation of afatinib and pembrolizumab combination therapy. Median values with 75th and 25th percentile whiskers. A mixed effects model was used to calculate the repeated measures *p* value. (*B*) Cell ratios of individual patients at baseline presented as column dot plots, PDL2^+^ monocytes to PDL2^+^ MDSC, CD4^+^ to CD8^+^ T-cells, and CD4^+^ to CD8^+^ central memory T-cells. Horizontal lines represent median values. (*C*) Receiver operator characteristic curve for baseline ratios of PDL2^+^ monocytes to PDL2^+^ MDSC cells, CD4^+^ to CD8^+^ central memory T-cells, and CD4^+^ to CD8^+^ T-cells. Corresponding AUCs of 0.93, 1.00, and 1.00 reveal the utility of cell ratios at baseline as predictors of antitumor activity of therapy. AUC, area under the curve; CD, cluster of differentiation; CM, central memory; MDSC, myeloid-derived suppressor cell; mono, monocyte; PDL2, programmed death-ligand 2.

When compared with nonresponders, there was a significant increase in the ratio of PD-L2+ monocytes to PD-L2+ myeloid-derived suppressor cells (MDSCs) in responders (and patients with prolonged PFS) (*p* <0.0001, mixed effects model, Fig. 4). This ratio at baseline was highly significant (*p* < 0.0001) and was excellent to discriminate between responders (and prolonged PFS) and nonresponders (AUC = 1.0). In addition, the ratio of CD4+ to CD8+ T-cells and the ratio of central memory CD4+ T-cells to central memory CD8+ T-cells were also significantly elevated in responders (and prolonged PFS) when compared with nonresponders (Fig. 4, *p* = 0.021 and <0.0001, respectively, mixed effects model). At baseline, these ratios were also significantly increased in responders (and prolonged PFS) (*p* = 0.042 and 0.00063, respectively, mixed effects model, Fig. 4) and could classify responders (and prolonged PFS) from nonresponders (AUC = 0.96 and AUC = 1, respectively, Fig. 4).

## Discussion

This study evaluated the immunomodulatory effects of the second-generation, irreversible EGFR TKI afatinib in combination with the PD-1 antibody pembrolizumab and included acquisition of baseline and on-treatment biopsies and peripheral blood samples at serial time points to evaluate alterations in circulating immune cells and the tumor immune microenvironment.

Our approach was limited by the changing landscape of treatment of EGFR-mutant NSCLC and modest antitumor activity observed (two of 11 PRs; 18% ORR). Though no DLTs were observed and the MTD was determined to be at the Food and Drug Administration–approved dose of afatinib (40 mg orally daily) and pembrolizumab (200 mg IV every 3 wk), irAEs occurred in four of 10 assessable patients (40%) (two grade 3 colitis, one grade 2 adrenal insufficiency, one grade 2 nephritis). Osimertinib is currently approved for first-line treatment of common EGFR-mutated NSCLC both as single agent and in combination with platinum-pemetrexed chemotherapy.^27^^,^^28^ Development of osimertinib and PD-(L)1 combination studies has been limited by high rates of pneumonitis. No pneumonitis events were observed with afatinib and pembrolizumab. Nevertheless, all four patients who had either a PR or PFS exceeding 6 months experienced grade 2 or more irAEs, further limiting administration of the combination. The LUX Lung IO study similarly evaluated afatinib in combination with pembrolizumab in advanced squamous NSCLC and was also halted due to irAEs and the changing landscape of treatment in advanced squamous NSCLC.^29^

Two patients had a PR to combination afatinib and pembrolizumab. One patient with PR had an acquired MET amplification as a putative mechanism of TKI resistance and PD-L1 TPS of 90% after progression on erlotinib. Another patient with a PR was a never smoker who had squamous histology EGFR exon 19 del NSCLC and PD-L1/PD-L2 co-amplification on NGS with PD-L1 TPS of 40%. PD-L1 amplification on NGS has been noted to be a predictive marker of benefit to PD-(L)1 blockade across multiple tumor types.^30^ The clinical activity of afatinib and pembrolizumab observed in select patients highlights that despite the overall lack of activity from PD(L)1 blockade in EGFR-mutated NSCLC, there are potential subsets of patients with EGFR-mutant lung cancer who may benefit from an immunotherapy-based approach. Molecular and immune studies to better identify predictors of benefit in exceptional responders to immunotherapy-based approaches in nonsmoking-associated oncogene-driven lung cancers is an area of unmet need.

Preclinical data in EGFR-mutant genetically engineered mouse models (GEMM) by Akbay et al.^8^ described the up-regulation of the PD(L)1 pathway in EGFR-driven lung cancers as a means of immune evasion that could be modulated by EGFR TKI. These results suggested the potential use of PD-1/PD-L1 inhibitors for the treatment of EGFR-mutant NSCLC and the potential benefits of adding EGFR TKI to PD(L)1. Nevertheless, multiple clinical trials of immunotherapy and first- to third-generation EGFR TKI combinations revealed no evidence for synergistic efficacy and safety profiles that limited clinical development. In our current trial, we observed some evidence of efficacy, but this was associated with toxicity.

A high throughput immune-oncology screen identified EGFR TKI as potent enhancers of antigen-specific cytotoxic T-cell–mediated tumor cell killing.^20^ Specifically, the investigators observed that afatinib was superior to other targeted therapies including other EGFR TKIs at enhancing T-cell killing and noted increased IFN-γ–induced MHC-Class I expression. Accompanying mechanistic studies to our trial revealed an increase in intratumoral CD3+ T-cells and a decrease in tumor Ki-67 expression in the patients who experienced prolonged stable disease/response, evidence that this treatment regimen may produce immunostimulatory antitumor responses.

We performed also high-parameter flow cytometry to determine the effects of afatinib and pembrolizumab combination therapy on the circulating immune cells, which yielded various interesting findings. For example, NK and NKT cells are immune populations with well-characterized anticancer properties,^31^ and flow cytometry revealed that both NK and NKT cells increased in response to concurrent afatinib and pembrolizumab (Fig. 2), which supports the ability of EGFR inhibition to alter NK cell function. These findings are relevant because previous preclinical studies revealed the ability of EGFR TKI to alter NK cell activity, although results were mixed. In one study, gefitinib attenuated NK cell-mediated lysis of tumor cells,^32^ whereas in another study, gefitinib enhanced NK cell-mediated cytotoxicity.^33^^,^^34^

Elevated co-expression of TIM3 and PD-1 are hallmarks of exhausted/dysfunctional T cells,^35^, ^36^, ^37^, ^38^ which have a reduced capacity to kill malignant cells. Bim has also been found to down-regulate CD8+ T-cell responses, especially in the setting of antigen persistence.^39^ In general, the tumor microenvironment is believed to promote T-cell exhaustion, evident by increased expression of inhibitory receptors and dysregulated expression of effector/activation markers and pore-forming proteins by CD8+ T-cells. It is therefore noteworthy that afatinib and pembrolizumab combination therapy significantly reduced circulating Bim+Tim3+PD1+CD11a+ CD8+ T-cells, which represent highly exhausted T-cells^39^^,^^40^ (Fig. 2).

Flow cytometry also identified candidate predictors of response to therapy. Specifically, patients who experienced disease control (and irAEs) had a distinct baseline circulating immune profile that included an elevated CD4-CD8 T-cell ratio in their peripheral circulation (Fig. 4). When peripheral blood CD4 and CD8 subpopulations were further characterized, it was discovered that the ratio of central memory CD4+ T-cells to central memory CD8+ T-cells was the best negative predictor of response to therapy (Fig. 4). Thus, a higher percentage of central memory CD8+ T-cells was associated with worse treatment responses (Figs. 3 and 4). Although this might seem counterintuitive, there was an inverse relationship between central memory and effector memory T-cells, that is, patients with lower central memory CD8+ T-cells tended to have elevated effector memory CD8+ T-cells. Thus, our findings are consistent with previous studies revealing that effector memory antigen-specific CD8+ T-cells can predict response to immunotherapy.^41^ A higher CD4/CD8 ratio in the tumor microenvironment was also noted in patients whose tumors had antitumor activity to afatinib/pembrolizumab, but this was not statistically significant (*p* = 0.4) with a small sample size (Supplementary Fig. 3).

Finally, there have been many reports revealing the tumor-promoting effects of MDSCs. Specifically, these cells suppress T-cell responses.^42^ Our study reveals that the ratio of monocytes to MDSCs in the peripheral circulation predicts response to therapy, that is, patients with a high monocyte to MDSC ratio (and thus relatively low levels of MDSCs) responded well to therapy (Fig. 4). Such patients started therapy with an intrinsically lower capacity to inhibit T-cells. Although this baseline immunophenotype predicted response to therapy, the immunomechanism of afatinib and pembrolizumab combination therapy involves an expansion of NK and NKT cells and a contraction of exhausted CD8 T-cells, as discussed earlier.

Overall, EGFR-mutated NSCLC is less responsive to immune checkpoint blockade than classic smoking-associated lung cancer. There is a large unmet need to optimize immunotherapy strategies in EGFR-mutant lung cancer to improve patient outcomes. Here, we revealed the feasibility of serial sampling of tissue and blood to detect immune cell alterations in the tumor microenvironment and in circulation that may underlie antitumor activity and toxicity with EGFR-directed therapy plus PD(L)1 inhibition. Limitations of this study include its small sample size, the exploratory nature of the biomarker studies, and the lack of monotherapy control groups.

Though combining EGFR TKI plus PD(L)1 inhibitors does not warrant further development in EGFR-mutated lung cancer due to increased AEs outweighing the clinical activity observed, EGFR-directed therapy with EGFR antibodies such as cetuximab does not seem to exhibit the same toxicity and has been found to have signals of clinical activity when combined with PD(L)1 blockade.^43^ Future studies evaluating immunotherapy in combination with other EGFR-directed therapies that may have less side effects when combined with PD-1 blockade such as the bispecific EGFR-MET antibody amivantamab or other next-generation EGFR antibodies are warranted.

## CRediT Authorship Contribution Statement

**Jonathan W. Riess:** Conceptualization, Data curation, Formal analysis, Funding acquisition, Investigation, Methodology, Project administration, Resources, Supervision, Validation, Roles/Writing - original draft, Writing - review and editing, Approval of Final Version.

**Matthew S. Lara:** Formal analysis, Investigation, Methodology, Project administration, Roles/Writing - original draft, Writing - review and editing, Approval of Final Version.

**Miguel Lopez de Rodas:** Data curation, Formal analysis, Investigation, Methodology, Project administration, Roles/Writing - original draft, Writing - review and editing, Approval of Final Version.

**Guillaume Luxardi:** Data curation, Formal analysis, Investigation, Methodology, Writing - review and editing, Approval of Final Version.

**Samantha Herbert:** Data curation, Formal analysis, Investigation, Methodology, Writing - review and editing, Approval of Final Version.

**Michiko Shimoda:** Data curation, Formal analysis, Investigation, Methodology, Writing - review and editing, Approval of Final Version.

**Karen Kelly:** Data curation, Writing - review and editing, Approval of Final Version.

**Alexander Merleev:** Data curation, Formal analysis, Investigation, Methodology, Writing - review and editing, Approval of Final Version.

**Elizabeth Moore:** Data curation, Writing - review and editing, Approval of Final Version.

**Laurel Beckett:** Formal analysis, Investigation, Methodology, Writing - review and editing, Approval of Final Version.

**Arta Monjazeb:** Methodology, Writing - review and editing, Approval of Final Version.

**Kurt Schalper:** Data curation, Formal analysis, Investigation, Methodology, Roles/Writing - original draft, Writing - review and editing, Approval of Final Version.

**Emanual Maverakis:** Data curation, Formal analysis, Investigation, Methodology, Roles/Writing - original draft, Writing - review and editing, Approval of Final Version.

**David R. Gandara:** Conceptualization, Data curation, Methodology, Writing - review and editing, Approval of Final Version.

## Disclosure

Dr. Riess reports receiving grants or contracts from ArriVent, Merck, Novartis, Nuvalent, Revolution Medicines, Kinnate, AstraZeneca, IO Biotech, Summit, Seattle Genetics; consulting fees from Novartis, Janssen, Catalyst, Biodesix, Amgen, Seattle Genetics, Merck, BMS, Beigene, Regeneron, Sanofi, Turning Point, Bayer, Merus NV, Daiichi Sankyo, OncoHost and support for attending meetings and/or travel from IO Biotech and AstraZeneca. Dr. Monjazeb reports grants or contracts from Merck, BMS, Transgene, Genentech, Incyte, Trisalus, IO Biotech, receipt of equipment, materials, drugs, medical writing, gifts or other services from Merck, Transgene and Incyte. Dr. Gandara reports consulting fees from Merck. Dr. Schalper reports receiving grants or contracts from Akoya Biosciences; AstraZeneca; Boehringer Ingelheim; Bristol Myers Squibb; F. Hoffmann-La Roche; Lilly; Merck Sharpe & Dohme; Ribon Therapeutics; Surface Oncology; Takeda Pharmaceutical Company Limited; and Tesaro Inc./GSK; consulting fees from AbbVie Inc.; Agenus Inc.; AstraZeneca; Bristol Myers Squibb; CDR-Life Inc.; Clinica Alemana de Santiago; EMD Serono Inc.; F.Hoffmann-La Roche; Genmab A/S; Indaptus Therapeutics; Janssen Pharmaceuticals, Inc.; Merck Sharpe & Dohme; Moderna, Inc.; Molecular Templates, Inc.; OnCusp Therapeutics; Parthenon/Incendia Therapeutics; Repertoire Therapeutics; Sanofi; Sensei Biotherapeutics; Shattuck Labs, Inc.; and Takeda Pharmaceutical Company Limited; payment or honoraria for lectures, presentations, speakers bureaus, manuscript writing or educational events from Bristol Myers Squibb; Fluidigm Corporation; Genmab A/S; Merck & Co., Inc.; Sanofi; PeerView and Takeda Pharmaceutical Company Limited and payment for expert testimony from AstraZeneca.
