# Supplementary material for: Immune Cell Dynamics in EGFR-Mutated NSCLC Treated With Afatinib and Pembrolizumab: Results From a Phase IB Study
Source: JTO Clin Res Rep. 2024 Jul 14;5(10):100706. doi: 10.1016/j.jtocrr.2024.100706 (PMC11420451; doi:10.1016/j.jtocrr.2024.100706)
Supplement: Supplementary file 2 [file mmc2.docx]

Supplemental Figure 2

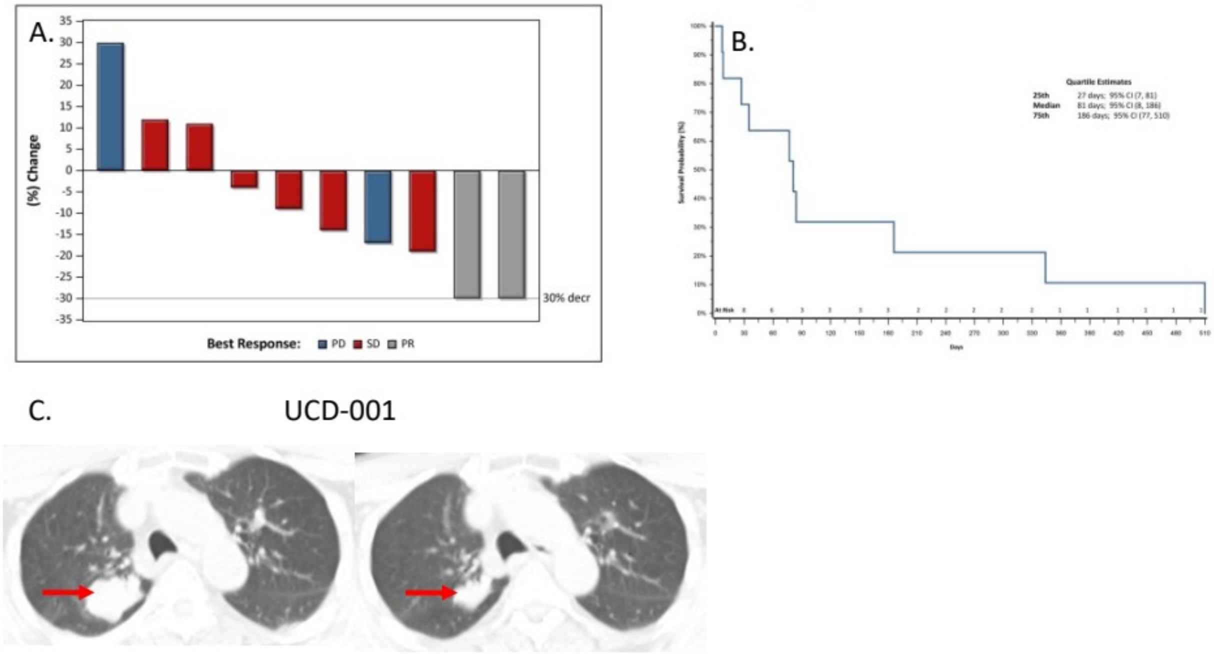

Baseline and Week 12 Scan with PR to treatment.
PD-L1 (22C3 40%) and PD-L1 and PD-L2 amp on NGS
