# Supplementary material for: Immune Cell Dynamics in EGFR-Mutated NSCLC Treated With Afatinib and Pembrolizumab: Results From a Phase IB Study
Source: JTO Clin Res Rep. 2024 Jul 14;5(10):100706. doi: 10.1016/j.jtocrr.2024.100706 (PMC11420451; doi:10.1016/j.jtocrr.2024.100706)
Supplement: Supplementary file 3 [file mmc3.docx]

Supplemental Figure 3

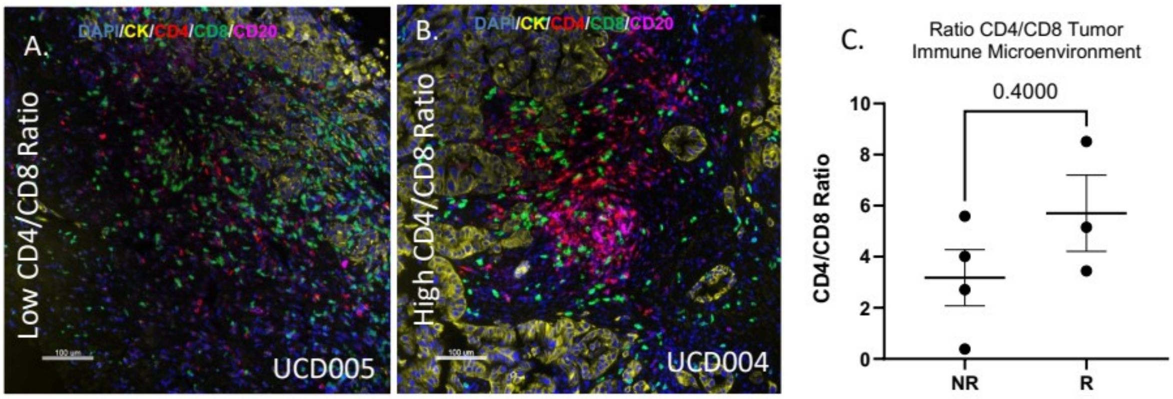

Pt with Hyperprogression

Pt with PFS > 6 mo.
